# Evaluating the cognitive effects of donepezil 23 mg/d in moderate and severe Alzheimer’s disease: analysis of effects of baseline features on treatment response

**DOI:** 10.1186/1471-2318-13-56

**Published:** 2013-06-06

**Authors:** Marwan Sabbagh, Jeffrey Cummings, Daniel Christensen, Rachelle Doody, Martin Farlow, Liang Liu, Joan Mackell, Randi Fain

**Affiliations:** 1 The Cleo Roberts Center for Clinical Research, Banner Sun Health Research Institute, 10515 W. Santa Fe Drive, Sun City, AZ 85351, USA; 2 Cleveland Clinic Lou Ruvo Center for Brain Health, Las Vegas, NV, USA; 3 University of Utah, Salt Lake City, UT, USA; 4 Baylor College of Medicine, Houston, TX, USA; 5 Department of Neurology, IU Alzheimer’s Disease and Related Disorders, Indianapolis, IN, USA; 6 Eisai Inc., Woodcliff Lake, NJ, USA; 7 Pfizer Inc., New York, NY, USA

**Keywords:** Alzheimer’s disease, Cognitive dysfunction, Donepezil, Severe impairment battery (SIB)

## Abstract

**Background:**

Treatment of Alzheimer’s disease with acetylcholinesterase inhibitors can result in symptomatic benefits, but patients often show variable responses. The objective of this post hoc analysis was to investigate relationships between easily identifiable baseline characteristics/demographics and cognitive response in patients treated with either donepezil 23 mg/d or 10 mg/d and to identify factors potentially influencing response.

**Methods:**

A post hoc analysis was conducted using data from a large, 24-week, randomized, double-blind, international study enrolling patients with moderate to severe Alzheimer’s disease (baseline Mini-Mental State Examination [MMSE], 0-20) (NCT 00478205). Cognitive changes in subgroups of patients based on selected baseline and demographic characteristics were compared using the least squares mean changes in Severe Impairment Battery scores at Week 24. Univariate and multivariate analyses were also performed.

**Results:**

Donepezil 23 mg/d provided statistically significant incremental cognitive benefits over donepezil 10 mg/d irrespective of baseline functional severity, measured by scores on the Alzheimer’s Disease Cooperative Study-Activities of Daily Living-severe version (*P* < 0.05). When patients were categorized by baseline cognitive severity (MMSE score), significant benefits of donepezil 23 mg/d over 10 mg/d were seen in both subgroups when based on MMSE scores of 0-9 versus 10-20 (*P* < 0.02 and *P* < 0.01, respectively), and in the more severe subgroup when based on MMSE scores of 0-16 versus 17-20 (*P* < 0.0001 and *P* > 0.05). Statistically significant incremental cognitive benefits of donepezil 23 mg/d over 10 mg/d were also observed regardless of age, gender, weight, or prestudy donepezil 10 mg/d treatment duration (*P* < 0.05). In the multivariate analysis, the only significant interaction was between treatment and baseline MMSE score.

**Conclusions:**

The cognitive benefits of donepezil 23 mg/d over 10 mg/d were achieved regardless of the patient’s age, gender, weight, duration of prior donepezil 10 mg/d, and functional severity. The influence of baseline cognitive severity on response seemed to be dependent on the level of impairment, with cognitive benefits of donepezil 23 mg/d over 10 mg/d most apparent in those patients at a more advanced stage of disease. These data may be useful in helping practicing physicians make informed decisions for their patients with advanced Alzheimer’s disease.

## Background

Donepezil is a reversible and highly selective acetylcholinesterase inhibitor (AChEI) that is approved for the symptomatic treatment of Alzheimer’s disease (AD). In numerous clinical trials since 1997, donepezil 5 and 10 mg/d has been shown to provide clinical benefits, with acceptable tolerability in the core symptom domains of cognition, global functioning, and function in activities of daily living [1]. Although both 5 and 10 mg doses have been shown to be effective in AD, an early systematic review of donepezil treatment and several subsequent clinical trials have indicated a relationship between benefits in cognition and higher donepezil dose [2-5]. Moreover, the cognitive benefits of a higher donepezil dose seem to be most prominent at more advanced stages of AD [4,5].

In 2010, a higher daily dose of donepezil (23 mg) was approved by the US Food and Drug Administration for the treatment of moderate to severe AD. This approval was based on results from a large phase 3 clinical trial comparing the 23 mg/d donepezil dose with the standard 10 mg/d donepezil dose [6]. In that trial, donepezil 23 mg/d provided statistically significant cognitive benefits over those achieved with donepezil 10 mg/d, as measured by the Severe Impairment Battery (SIB). On the co-primary measure of global function (the Clinician’s Interview-Based Impression of Change-plus caregiver input [CIBIC-plus]), no significant benefit was observed with higher-dose versus standard-dose donepezil.

Although symptomatic benefits have been widely reported with donepezil and other AChEIs, treated patients typically respond variably. For example, in a pooled analysis of data from studies of donepezil 10 mg/d in patients with severe AD, 66.8% of donepezil-treated patients showed stabilized or improved scores on the SIB; however, in approximately one quarter of these “responding” patients, SIB scores were stabilized or improved less than 4 points, whereas in around one third of patients, SIB scores had improved by at least 12 points [7]. A number of studies have investigated whether candidate factors, such as patient age, the rate of pretreatment decline, the extent of medial temporal lobe atrophy, and the level of cognitive impairment [8-11] might influence response to AChEI treatment among patients with AD.

Two specific post hoc analyses have previously been performed. These examined the influence of memantine use and geographic region (US versus non-US subgroups) on respective efficacy outcomes with donepezil 23 mg/d and 10 mg/d using data from the clinical trial comparing the 2 donepezil doses [12,13]. In these analyses, donepezil 23 mg/d provided cognitive benefits over donepezil 10 mg/d, regardless of concomitant memantine use and in both the US-based and non-US-based subgroups, indicating that memantine use and geographic location appear to have no direct substantial influence over the cognitive benefits of higher-dose versus standard-dose donepezil [12,13]. The objective of the current analysis was to further investigate relationships between other selected baseline characteristics/ demographics (i.e., characteristics/demographics not previously studied in post hoc analysis) and cognitive response in patients receiving either donepezil 23 mg/d or donepezil 10 mg/d and to identify factors potentially influencing response. Since the original study compared 2 active therapies for AD, this analysis focused on the possible influence of these baseline factors on incremental benefits of higher-dose donepezil over standard-dose donepezil. Better understanding of easily identifiable factors influencing response to donepezil 23 mg/d versus 10 mg/d would be useful for generating hypotheses that, if confirmed, could provide treating physicians with information relevant to clinical decision-making in this patient population.

## Methods

### Study design

A comprehensive description of the original trial methods has been published previously [6]. Briefly, this 24-week, randomized, parallel-group, double-blind, multinational trial enrolled patients with a diagnosis of probable AD with a Mini-Mental State Examination (MMSE) score of 0 to 20 (moderate to severe) and a SIB score of ≤ 90. Eligible patients had been receiving donepezil 10 mg/d for at least 3 months prior to screening. Patients were randomized in a 2:1 ratio to transition to donepezil 23 mg/d or to continue treatment with donepezil 10 mg/d (see Additional file 1). Patients were eligible if taking daily doses of ≤ 20 mg memantine for at least 3 months prior to screening. Patients were stratified by concomitant use of memantine, but memantine was not considered a study medication and was not provided to patients during the trial, nor was there monitoring of adherence. Primary efficacy end points were SIB total score change from baseline to Week 24 and the CIBIC-plus overall change score at Week 24. Secondary efficacy end points included Alzheimer’s Disease Cooperative Study-Activities of Daily Living-severe version (ADCS-ADL-sev) and MMSE score changes from baseline to Week 24. Before conducting study procedures, investigators obtained written informed consent from each patient, if possible, or from the patient’s legal guardian or representative. If a patient was unable to provide written consent, verbal assent was required for participation, and a caregiver was required to provide separate written informed consent in the study. The protocol and informed consent form for the original study were approved by the independent ethics committee/institutional review board for each research site and conformed to the principles of the World Medical Association Declaration of Helsinki and all local regulations. The study design was reviewed and deemed appropriate by the FDA and other regulatory agencies.

### Relationship of response to baseline features

A post hoc analysis was conducted to evaluate the relationships between selected baseline patient characteristics or demographics and cognitive response in patients treated with either donepezil 23 mg/d or donepezil 10 mg/d. All subgroup comparisons consisted of comparing the differences between least squares (LS) mean changes from baseline. Least squares mean changes in SIB scores from baseline to Week 24 (last observation carried forward [LOCF]) were analyzed for subgroups of patients based on baseline patient and demographic characteristics, including age, gender, weight, prestudy donepezil 10 mg/d treatment duration, baseline cognitive severity (MMSE score), and baseline functional severity (ADCS-ADL-sev score).

For age, weight, prestudy donepezil 10 mg/d treatment duration, and baseline functional severity, subgroups were defined on values above or below the overall median value. The gender subgroups were male versus female patients. Finally, the baseline cognitive severity subgroups were based on either traditional severe versus moderate subpopulations (MMSE 0-9 vs MMSE 10-20) or on patients considered to have more or less severe cognitive impairment (MMSE 0-16 vs MMSE 17-20) as categorized in the original study publication [6]. This second MMSE categorization was also used to provide an insight into the relative cognitive effects of the 2 donepezil doses in patients with moderate to severe AD (MMSE 0-16) compared with those with mild to moderate disease (MMSE 17-20).

Univariate and multivariate analyses were also performed using data from the intent-to-treat (ITT) population (all patients who received at least 1 dose of study medication and who had available baseline and postbaseline data for at least 1 of the 2 co-primary end points). For the purposes of these analyses, “response” was defined as a change from baseline in SIB total score (Week 24 LOCF); this was the absolute change in SIB and, in this case, does not refer to a therapeutic response of one donepezil dose over the other. Variables in the models included the baseline characteristics studied in the current subgroup assessments—treatment (23 mg/d vs 10 mg/d), age (continuous), gender (male vs female), weight (continuous), prestudy donepezil 10 mg/d treatment duration (continuous), baseline MMSE score (continuous), and baseline ADCS-ADL-sev score (continuous)—as well as the previously assessed baseline characteristics: concomitant memantine use (yes vs no) and geographic region (US vs non-US) [12,13]. Due to a significant positive correlation between baseline MMSE score and ADCS-ADL-sev score and the resultant potential for multicollinearity, baseline ADCS-ADL-sev score was omitted from the multivariate model. Significance was measured at the 0.05 level.

## Results

### Overall trial results

#### Patients

In total, 1467 patients were randomized to donepezil treatment and the ITT population was 1371. Of these, 909 patients received donepezil 23 mg/d and 462 received donepezil 10 mg/d. Demographics and mean baseline disease characteristics were similar for both treatment groups. Relevant baseline data for the ITT population are shown in Table 1.

**Table 1:** **Demographics and baseline characteristics of the intent**-**to**-**treat population**

**Characteristic**	**Donepezil 23 mg**/**d**	**Donepezil 10 mg**/**d**
Age, years		
Number of patients	909	462
Mean (± SD)	73.8 (8.48)	73.8 (8.55)
Median	75.0	75.0
Range	47-89	49-90
Gender		
Number of patients	909	462
Males, n (%)	335 (36.9)	175 (37.9)
Females, n (%)	574 (63.1)	287 (62.1)
Weight, kg		
Number of patients	908	462
Mean (± SD)	66.7 (14.8)	66.2 (14.4)
Median	65.5	64.5
MMSE		
Number of patients	908	462
Mean (± SD)	13.1 (4.99)	13.1 (4.72)
Median	14.0	14.0
ADCS-ADL-sev		
Number of patients	908	461
Mean (± SD)	34.1 (10.88)	34.5 (11.19)
Median	36.0	36.0
SIB		
Number of patients	907	462
Mean (± SD)	74.2 (17.58)	75.6 (16.28)
Median	81.0	82.0
CIBIS-plus		
Number of patients	904	461
Mean (± SD)	4.42 (0.85)	4.38 (0.89)
Median	4.0	4.0
Duration of prestudy		
donepezil 10 mg/d, weeks		
Number of patients	909	462
Mean (±SD)	113.4 (108.4)	104.9 (99.2)
Median	71.9	66.3

MMSE = Mini-Mental State Examination; ADCS-ADL-sev = Alzheimer’s Disease Cooperative Study-Activities of Daily Living Inventory, severe version; SIB = Severe Impairment Battery; CIBIS-plus = Clinician’s Interview-Based Impression of Severity-plus caregiver input; SD = standard deviation.

### Post hoc analysis: impact of disease characteristics

**Figure 1:**
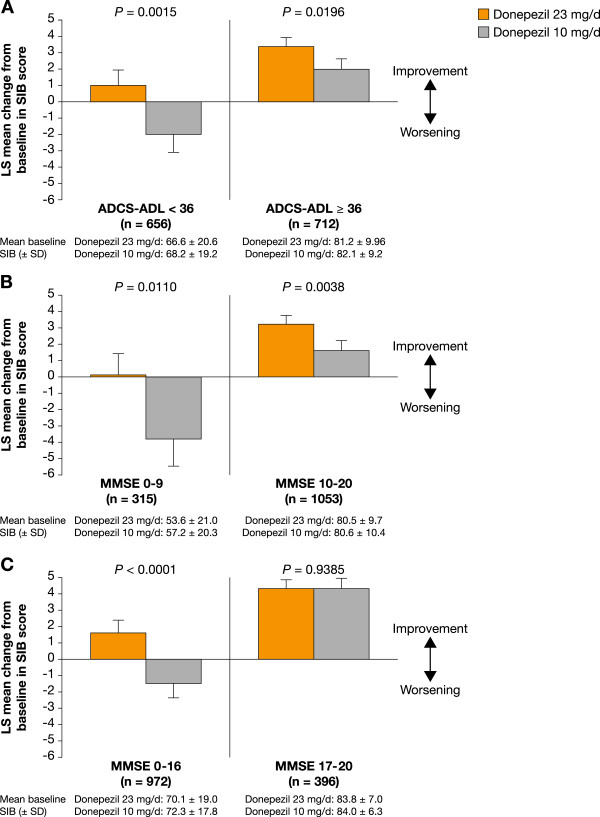
**Effect of disease characteristics on the efficacy of donepezil 23 mg**/**d or 10 mg**/**d measured by LS mean change in SIB score from baseline to Week 24. A**. Functional level. **B**. Severity, MMSE 0-9 or 10-20. **C**. Severity, MMSE 0-16 or 17-20.

Donepezil 23 mg/d provided statistically significant incremental cognitive benefits (*P* < 0.05 for both subgroups) over donepezil 10 mg/d irrespective of baseline functional severity (Figure 1A).

In patients with lower functional abilities at baseline (below the median baseline ADCS-ADL-sev score), SIB scores increased with donepezil 23 mg/d (LS mean change: +1.0 point) but declined with donepezil 10 mg/d (LS mean change: -2.0 points). However, in patients with greater functional abilities at baseline (above the median baseline ADCS-ADL-sev score), SIB scores increased with both donepezil doses but the magnitude of the increase was greater with donepezil 23 mg/d than with donepezil 10 mg/d (LS mean change: +3.4 vs +2.0 points).

When baseline cognitive severity was assessed based on traditional MMSE scores for severe and moderate disease (MMSE 0-9 vs 10-20), significant benefits of donepezil 23 mg/d over donepezil 10 mg/d were evident in both subgroups (*P* = 0.011 and *P* = 0.0038, respectively; Figure 1B). In the MMSE 0-9 subgroup, SIB scores were relatively stabilized with donepezil 23 mg/d (LS mean change: +0.1 points), but declined with donepezil 10 mg/d (LS mean change: -3.8 points) whereas in the MMSE 10-20 subgroup SIB scores increased with both donepezil doses but the magnitude of the increase was greater with donepezil 23 mg/d than 10 mg/d (LS mean change: +3.2 vs +1.6 points). When baseline cognitive severity was assessed based on more or less severe cognitive impairment (MMSE 0-16 vs 17-20) as categorized in the original study publication [6], significant benefits of donepezil 23 mg/d over donepezil 10 mg/d were observed for the more severe subgroup (*P* < 0.0001), but not for the less severe subgroup (Figure 1C). In the MMSE 0-16 subgroup, SIB scores increased with donepezil 23 mg/d (LS mean change: +1.6 points), but declined with donepezil 10 mg/d (LS mean change: -1.5 points); in the MMSE 17-20 subgroup, SIB scores increased with both donepezil doses (LS mean change: +4.3 points in both treatment groups).

### Post hoc analysis: impact of patient characteristics/demographics

**Figure 2:**
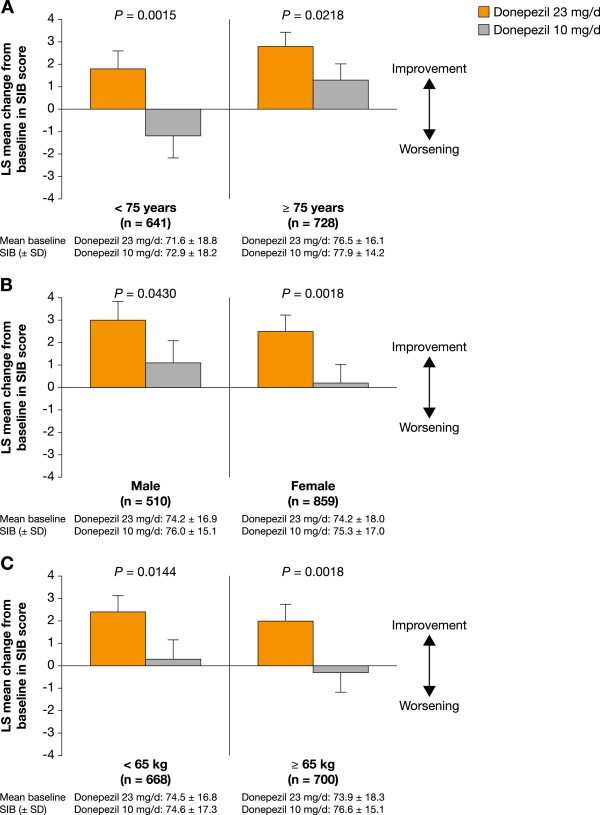
**Effect of patient characteristics**/**demographics on the efficacy of donepezil 23 mg**/**d or 10 mg**/**d measured by LS mean change in SIB score from baseline to Week 24. A**. Age. **B**. Gender. **C**. Weight.

Statistically significant incremental cognitive benefits of donepezil 23 mg/d over donepezil 10 mg/d were observed regardless of age, gender, or weight (all *P* < 0.05) (Figure 2A, B, and C). For the analysis based on age (Figure 2A), SIB scores increased with donepezil 23 mg/d (LS mean change: +1.8 points) in the lower age subgroup (< 75 years), but declined with donepezil 10 mg/d (LS mean change: -1.2 points). In the older age subgroup (≥ 75 years), SIB scores improved in both treatment arms, but the magnitude of the increase was greater with donepezil 23 mg/d than with donepezil 10 mg/d (LS mean change: +2.8 vs +1.3 points). For the analyses based on gender and weight, the pattern of cognitive response was relatively similar between the respective subgroups (Figure 2B and C).

### Post hoc analysis: impact of treatment characteristics

**Figure 3:**
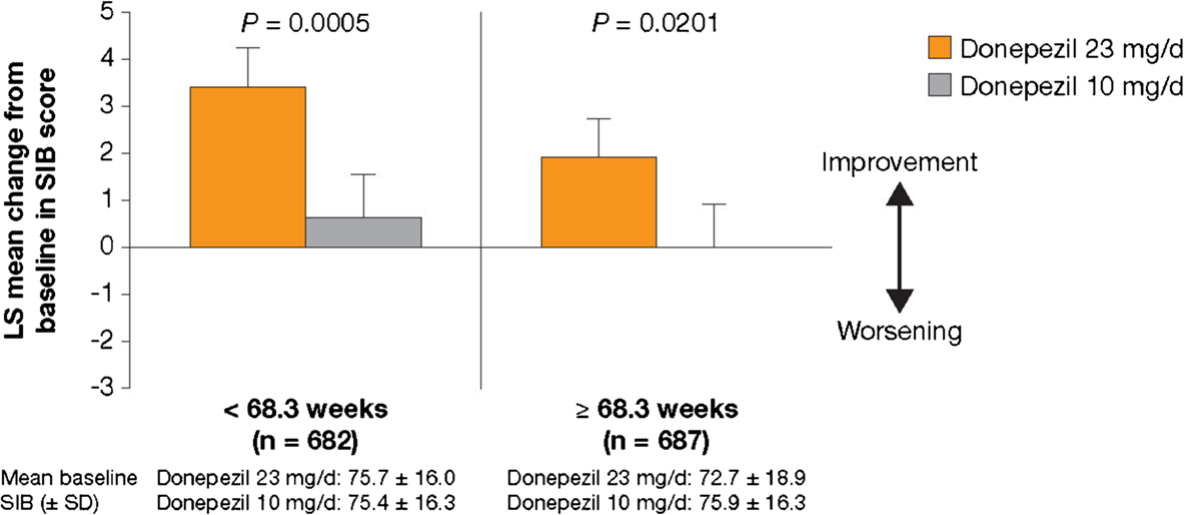
**Effect of prior 10 mg treatment duration on the efficacy of donepezil 23 mg**/**d or 10 mg**/**d measured by LS mean change in SIB score from baseline to Week 24.**

Statistically significant incremental cognitive benefits of donepezil 23 mg/d over 10 mg/d were observed regardless of prestudy donepezil 10 mg/d treatment duration (*P* < 0.05) (Figure 3).

The duration of prestudy donepezil 10 mg/d use (< 68.3 weeks vs ≥ 68.3 weeks) did not influence the magnitude of the effect when patients were subsequently treated with donepezil 23 mg/d; SIB scores increased with donepezil 23 mg/d (LS mean change: +3.4 points in the shorter duration subgroup and +1.9 points in the longer duration subgroup) but only marginally increased or stabilized with the donepezil 10 mg/d (LS mean change: ≤ +0.6 points in both subgroups; Figure 3).

### Post hoc analysis: univariate and multivariate analyses

In the univariate model, treatment with donepezil 23 mg/d, older age, not taking concomitant memantine, a higher baseline MMSE score, and a higher baseline ADCS-ADL-sev score were significantly associated with incremental changes in SIB score at Week 24 (*P* < 0.01). Significant interactions were identified between treatment and age (*P* = 0.0199) and between treatment and baseline MMSE score (*P* = 0.0013). In the multivariate model, treatment with donepezil 23 mg/d (*P* < 0.0001), higher baseline MMSE scores (*P* < 0.0001), and not taking concomitant memantine (*P* = 0.0434) were the only significant factors associated with larger increments in SIB score at Week 24. The significant interaction between treatment and baseline MMSE score was also retained in the multivariate model (*P* = 0.0013).

## Discussion

Results from this post hoc analysis suggest that the cognitive benefits reported from the original study of donepezil 23 mg/d versus donepezil 10 mg/d may be achieved regardless of a patient’s age, gender, weight, duration of prior donepezil 10 mg/d treatment, and functional severity. Patients transitioning to the higher dose experience significant cognitive benefits compared with those who continue treatment with 10 mg/d [6]. However, the current analyses also suggest that the cognitive benefits of donepezil 23 mg/d over 10 mg/d may be more evident in patients with more advanced cognitive and/or functional deficits.

In previous studies, patient age has shown a variable influence on response. For example, Evans et al. [8] found a significantly greater improvement in patients treated with donepezil who were aged ≤ 65 years (compared with age > 65 years), whereas Connelly et al. [9] found no effect of patient age on response to AChEI treatment. In the current study, age did not appear to influence the benefits of donepezil 23 mg/d over 10 mg/d, but younger patients showed worse overall responses compared with older patients in the dichotomous analysis and older age was significantly associated with SIB improvement in the univariate analysis. This observation could reflect different disease features and/or causality (genes, environmental factors) or an association of younger age with a more aggressive disease course (faster progression) [14-17]. Younger patients may also be more likely to possess the apolipoprotein E4 allele [18], which may influence cognitive decline [19].

Gender and weight had little effect on the response to higher-dose donepezil. Although results from this analysis suggest that patient weight may not be an influencing factor for the cognitive benefits of donepezil 23 mg/d, prior safety analyses have indicated that patients with very low body weight may be more likely to experience treatment-emergent adverse events upon transition to the higher donepezil dose. Patient weight must therefore be taken into account when considering the tolerability of donepezil 23 mg/d [6,20].

In terms of the studied treatment characteristics, the duration of prestudy treatment with 10 mg/d donepezil does not appear to influence the magnitude of effect of donepezil 23 mg/d versus 10 mg/d. Although not assessed in the subgroup analysis, concomitant memantine use was included as a variable in the statistical models and not taking memantine was independently associated with incremental changes in SIB score. While this outcome appears to contrast with previous data on the benefits of donepezil and memantine combination therapy [21], there are clear differences in study design and analysis methodology between the current and prior trials. Furthermore, the modeling results do not rule out baseline disease severity as an influencing factor in the relationship between memantine use and SIB response in patients receiving donepezil.

With respect to the assessed disease characteristics, patient subgroups with less severe baseline disease (higher ADCS-ADL-sev and MMSE scores) showed the greatest level of SIB improvement with both donepezil 23 mg/d and donepezil 10 mg/d. However, the results presented here indicate that the differential between the effects of donepezil 23 mg/d and donepezil 10 mg/d was greatest in patients with more severe disease (lower ADCS-ADL-sev and MMSE scores). Indeed, the cognitive benefits of donepezil 23 mg/d compared with donepezil 10 mg/d were most apparent in those patients who were at a more advanced stage of disease at study baseline. The suggestion that more severe patients may be more likely to respond to an increase in donepezil dose appears to be further supported by the univariate and multivariate models. As expected these models indicated that higher baseline MMSE scores are associated with incremental changes in SIB score, regardless of the donepezil dose; however, the presence of a significant interaction between treatment and baseline MMSE indicates that the influence of baseline MMSE on the change in SIB score during the study was significantly different between the donepezil 23 mg/d and 10 mg/d treatment groups.

There is a scientific rationale for why patients with more severe AD may obtain greater benefit from a daily dose of donepezil greater than 10 mg [22]. First, in patients receiving 10 mg/d of donepezil, the extent of acetylcholinesterase inhibition as measured by PET imaging is no greater than 40% [23,24]. Second, examination of brain tissue has demonstrated more pronounced cholinergic deficit in patients who have more severe clinical symptoms [25]. As a result, patients with more severe disease may require a greater amount of acetylcholinesterase inhibition in order to achieve an optimal degree of cholinergic stimulation, which a higher dose of donepezil may provide [22].

While the results of these post hoc analyses are consistent with the primary outcomes observed in the original study, they must be viewed cautiously. They were not separately powered for significance and their interpretation must be considered in view of the demographics chosen and particularly in light of clinical experience. Moreover, data on other characteristics, for example the rate of pre-treatment cognitive decline, were not available and therefore their influence on the SIB outcomes could not be assessed. Furthermore, the findings are based on post hoc subanalyses, which were not prospectively defined, and thus these observations can only generate exploratory hypotheses, which will require prospective confirmation.

## Conclusions

The findings of this post hoc analysis suggest that the cognitive benefits of donepezil 23 mg/d over donepezil 10 mg/d may be achieved regardless of a patient’s age, gender, weight, prior duration of donepezil 10 mg/d treatment, and functional severity, and that these incremental benefits may be more evident in patients with more advanced AD. Physicians faced with making decisions about the optimum treatment for their patients with AD need reliable information regarding timing and doses of treatment. The data from the current analyses may be useful for physicians and other prescribers treating patients with advanced AD, particularly when the illness is progressing despite treatment with donepezil 10 mg.

## Abbreviations

AChEI: Acetylcholinesterase inhibitor; AD: Alzheimer’s disease; ADCS-ADL-sev: Alzheimer’s Disease Cooperative Study-Activities of Daily Living-severe version; CIBIC-plus: Clinician’s Interview-Based Impression of Change-plus caregiver input; CIBIS-plus: Clinician’s Interview-Based Impression of Severity-plus caregiver input; ITT: intent-to-treat; LOCF: last observation carried forward; LS: Least squares; MMSE: Mini-Mental State Examination; SIB: Severe Impairment Battery.

## Competing interests

MS: Has served as a consultant/advisor to Amerisciences, BMS, Takeda, Bayer, Eisai, and Pfizer; he receives royalties from Wiley and Amerisciences, and has received contract/grant support from Avid, Lilly, Bayer, Baxter, GE, Pfizer, Janssen, BMS, and Eisai. JC: Has provided consultation to Abbott, Acadia, ADAMAS, Anavex, Astellas, AstraZeneca, Avanir, Baxter, Bristol-Myers Squibb, Eisai, Elan, EnVivo, Forest, Genentech, GlaxoSmithKline, Janssen, Lilly, Lundbeck, Medtronics, Merck, Neurokos, Neuronix, Novartis, Otsuka, Pain Therapeutics, Pfizer, Plexxicon, Prana, QR, Sanofi, Sonexa, Takeda, and Toyama pharmaceutical companies. He has also provided consultation on diagnostic assessment to Bayer, Avid, GE, MedAvante, Neurotrax, and UBC. Dr. Cummings owns stock in ADAMAS, Prana, Sonexa, MedAvante, Neurotrax, Neurokos, and QR pharma. He is a speaker/lecturer for Eisai, Forest, Janssen, Novartis, Pfizer, and Lundbeck. Dr. Cummings owns the copyright of the Neuropsychiatric Inventory, and has provided expert witness consultation regarding olanzapine and ropinirole. DC: Has received consultant fees from Pfizer, Eisai, and Medivation, and has been on the Speakers’ Bureau for Pfizer and Eisai; he holds stock in Pfizer, and Johnson & Johnson. RD: Has provided consultation to Accera, AC Immune, Allon, Astellas, Banner Health, Biote, Bristol MeyersSquibb, Cardeus, Chiesi, Comentis, Dainippon, Debiopharm, Elan, Epix, Forest, Genzyme, GlaxoSmithKline, Hoffman-LaRoche, Janssen, Lilly, Medivation Inc., Merck and Co. Inc., Merck-Serono, Novartis, Nutricia, Ocera, Ono, Otsuka, Pfizer, Prana, QR Pharma, Schering-Plough, Shire, Sonexa, Sanofi-Aventis, Suven, Takeda, Targacept, Transition, Varinel, and Zinfandel. Her institution has received grant/contract support for clinical trials from Elan, Genentech, Janssen, Myriad, Pfizer, and Wyeth. She holds stock options in QR Pharma, Sonexa and Transition. MF: Has served as a paid consultant for Accera, Alltech, Astellas, Bayer, Bristol-Myers Squibb, Eisai Medical Research, GE Healthcare, Helicon, Medavante, Medivation Inc.,Merck and Co. Inc., Novartis Pharma, Pfizer, Prana Biotech, QR Pharma, Sanofiaventis Groupe, Schering-Plough, Lilly, Shire Pharmaceuticals, and Toyama; is a paid speaker for Eisai, Forest, Novartis, and Pfizer; and receives research support from Eisai, Eli Lilly and Co., Genentech, Novartis Pharm., and Roche. LL: Serves as a statistician consultant to Eisai Inc. JM: Employee of Pfizer Inc. RF: Employee of Eisai Inc.

## Authors’ contributions

MS: Helped conceive/design the described analyses, participated in the data analysis, and assisted in the drafting, editing, and interpretation of the manuscript. JC: Helped conceive/design the described analyses, participated in the data analysis, and assisted in the drafting, editing, and interpretation of the manuscript. DC: Helped conceive/design the described analyses, participated in the data analysis, and assisted in the drafting, editing, and interpretation of the manuscript. RD: Helped conceive/design the described analyses, participated in the data analysis, and assisted in the drafting, editing, and interpretation of the manuscript. MF: Helped conceive/design the described analyses, participated in the data analysis, and assisted in the drafting, editing, and interpretation of the manuscript. LL: Statistically assisted in the planning and performance of the post-hoc analyses and assisted in the drafting, editing, and interpretation of the manuscript. JM: Helped conceive/design the described analyses, participated in the data analysis, and assisted in the drafting, editing, and interpretation of the manuscript. RF: Helped conceive/design the described analyses, participated in the data analysis, and assisted in the drafting, editing, and interpretation of the manuscript. All authors read and approved the final version of the manuscript.

## Pre-publication history

The pre-publication history for this paper can be accessed here:

http://www.biomedcentral.com/1471-2318/13/56/prepub

## Supplementary Material

Additional file 1 CONSORT 2010 Flow Diagram. Click here for file
