# Supplementary material for: Evaluating the cognitive effects of donepezil 23 mg/d in moderate and severe Alzheimer’s disease: analysis of effects of baseline features on treatment response
Source: BMC Geriatr. 2013 Jun 6;13:56. doi: 10.1186/1471-2318-13-56 (PMC3681558; doi:10.1186/1471-2318-13-56)
Supplement: Additional file 1 — CONSORT 2010 Flow Diagram. [file 1471-2318-13-56-S1.doc]

**CONSORT 2010 Flow Diagram**

**Allocation**

**Analysis**

**Follow-Up**

**Enrollment**

Assessed for eligibility (n=2186)

Excluded (n=719)

♦  Not meeting inclusion criteria (n=265)

♦  Declined to participate (n=59)

♦  Other reasons (n=395)

Analysed (n=963)
♦ Excluded from analysis (lack of post-baseline assessment, did not receive study drug) (n=18)

Lost to follow-up (adverse events, medical non-compliance, withdrew consent, protocol violation, request of investigator or sponsor, lack of efficacy, other) (n=296)

Discontinued intervention (medical non-compliance, withdrew consent) (n=70)

Allocated to Donepezil 23 mg (n=981)

♦ Received allocated intervention (n=972)

♦ Did not receive allocated intervention (discontinued study prior to receiving study drug) (n=9)

Lost to follow-up (adverse events, medical non-compliance, withdrew consent, protocol violation, request of investigator or sponsor, other) (n=87)

Discontinued intervention (medical non-compliance, withdrew consent) (n=26)

Allocated to Donepezil 10 mg (n=486)

♦ Received allocated intervention (n=479)

♦ Did not receive allocated intervention (discontinued study prior to receiving study drug) (n=7)

Analysed (n=471)
♦ Excluded from analysis (lack of post-baseline assessment, did not receive study drug) (n=15)

Randomized (n=1467)
